# Supplementary figures and images for: The evolutionary history of topological variations in the CPA/AT transporters
Source: PLoS Comput Biol. 2021 Aug 17;17(8):e1009278. doi: 10.1371/journal.pcbi.1009278 (PMC8396727; doi:10.1371/journal.pcbi.1009278)

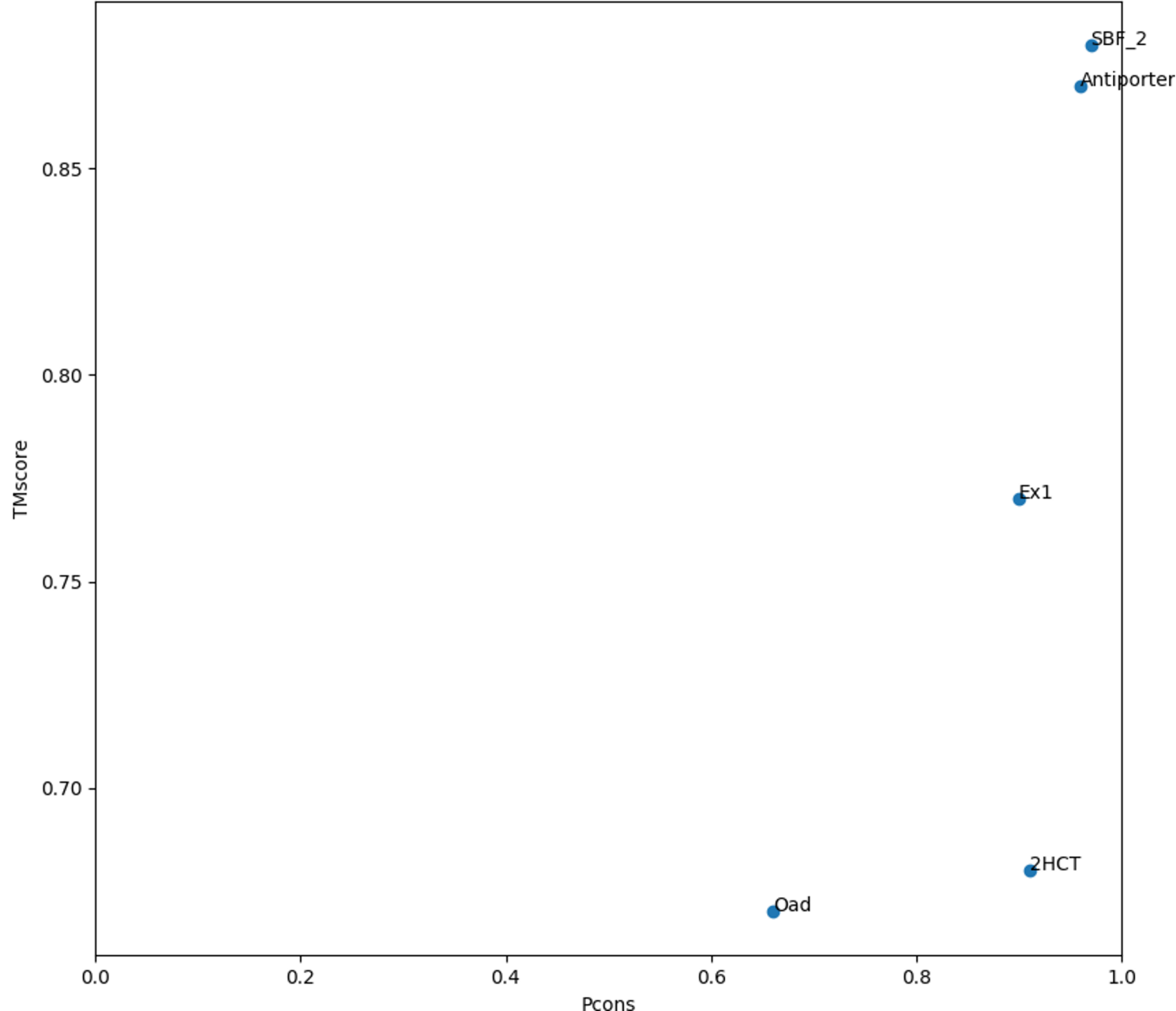

Supplement: S1 Fig — (PDF) [file pcbi.1009278.s001.pdf]

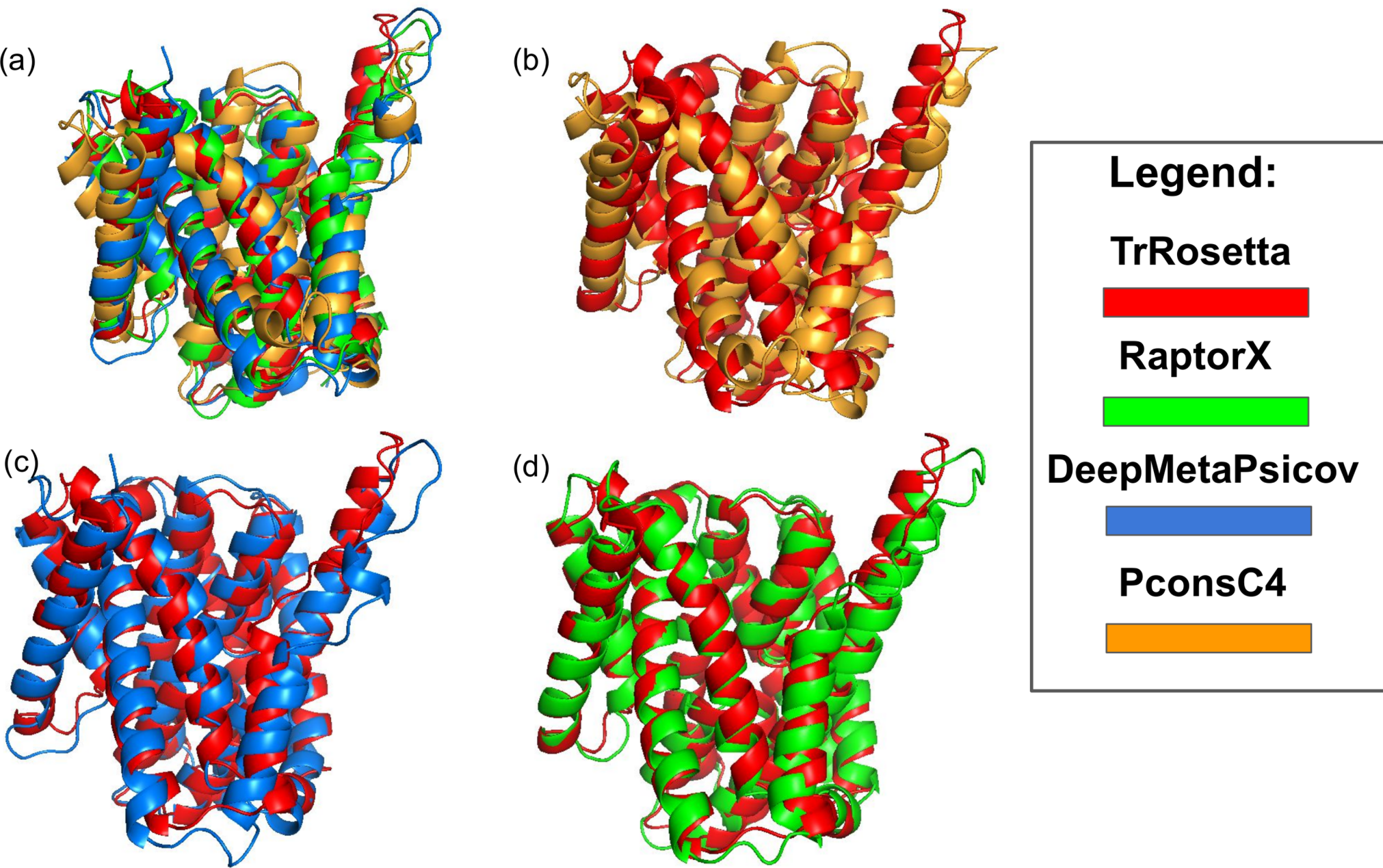

Supplement: S2 Fig — a) The superposition among the four. b) trRosetta vs PcosnC4 c) trRosetta vs DeepMetaPsicov d) trRosetta vs PconsC4 (PDF) [file pcbi.1009278.s002.pdf]

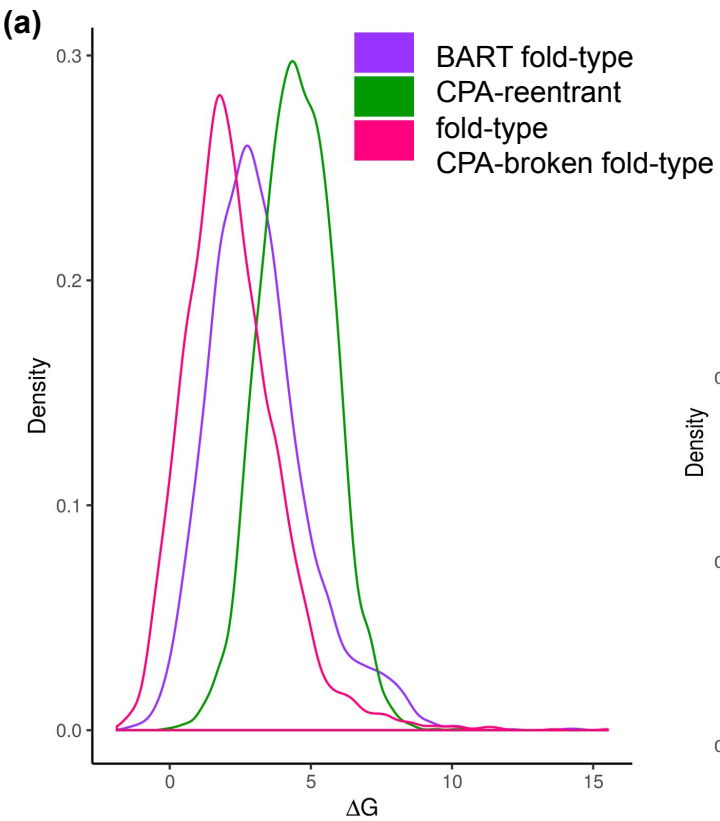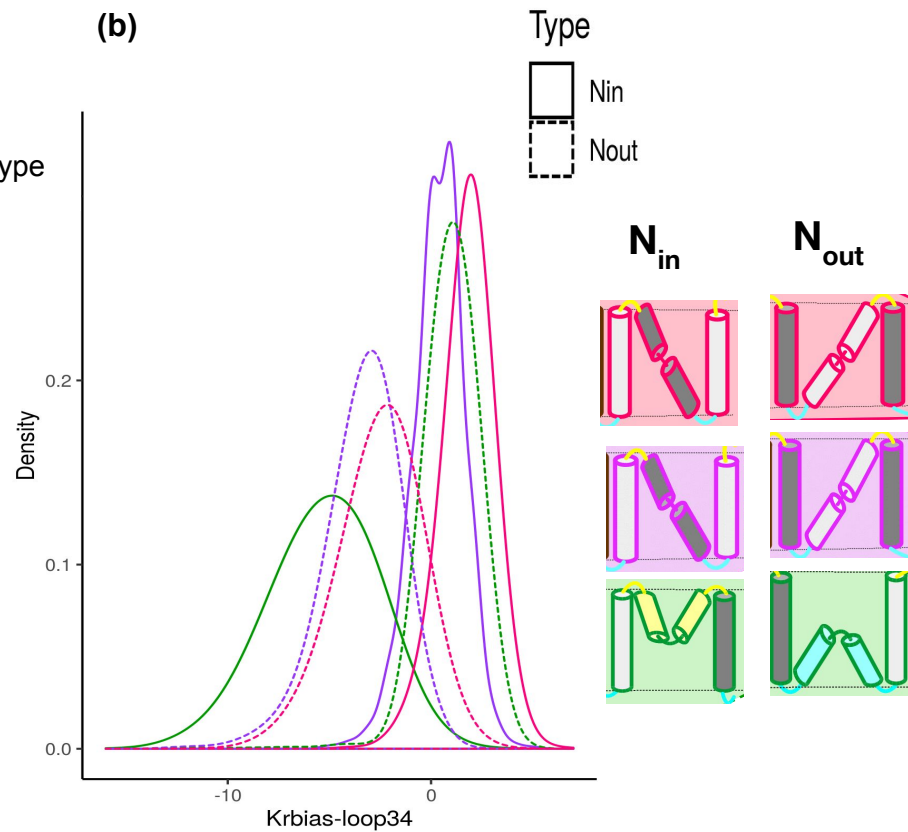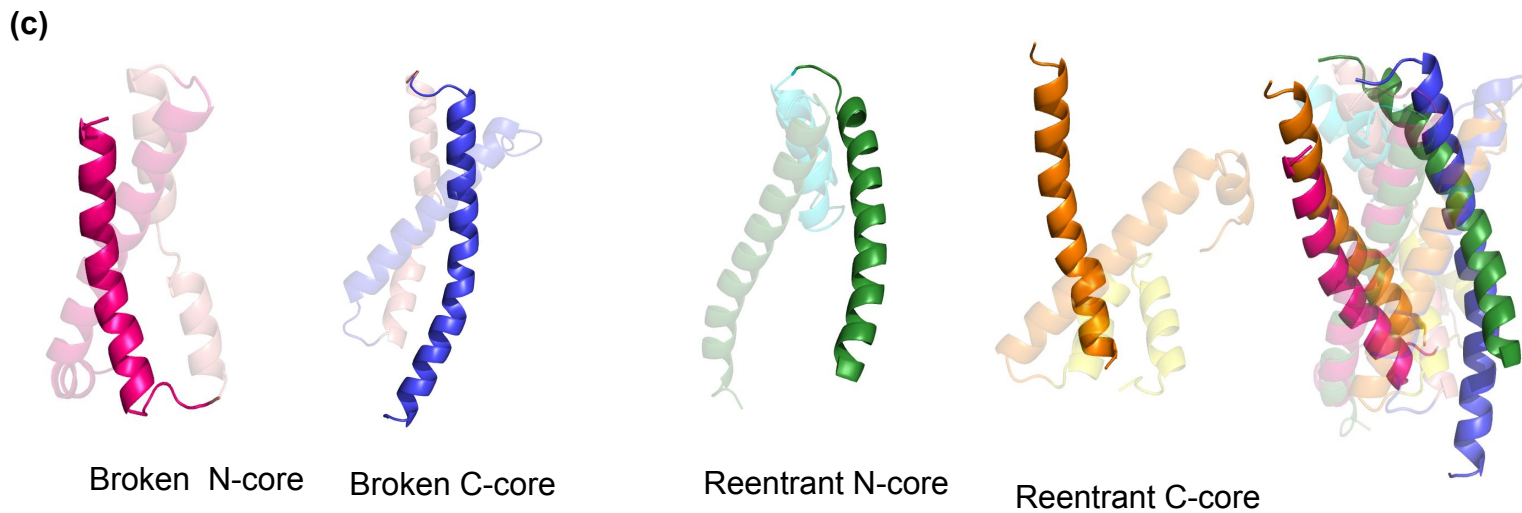

Supplement: S4 Fig — (a) Density plot showing the hydrophobicity of broken and reentrant helices belonging to the three fold-types. (b) Density plot for KR-bias of the last helix of the N- and C-terminal core subdomain belonging to three fold-types. Nin and Nout core subdomains of the three fold-types are shown as cartoon representations. The last helix shows the change in orientation. Nin and Nout denote the orientation of the first helix of the core subdomain. (c) The first four figures show the N- and C-terminal core subdomains of the broken and reentrant transporter. The last helix is shown dark while the other two helices are shown in transparent colour. The final figure shows the structure superimposition between the broken and reentrant core domains. (PDF) [file pcbi.1009278.s004.pdf]

**(a)** Glt\_Symporter

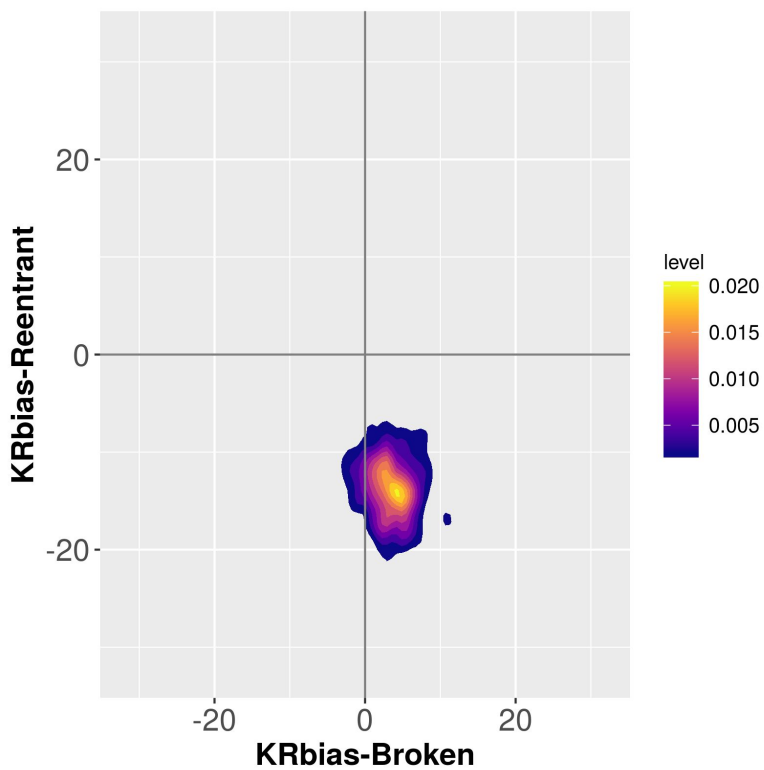

**(b)** AbrB

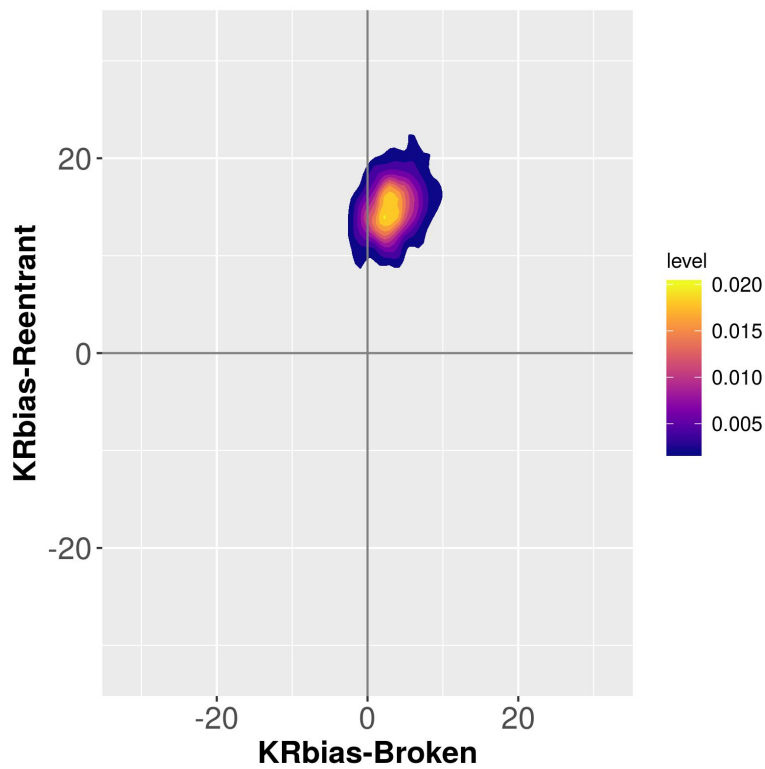

Supplement: S5 Fig — (a) KR bias plots showing the orientation of Glt_symporter. (b) KR bias plots showing the orientation of AbrB. (PDF) [file pcbi.1009278.s005.pdf]

(a) A:Mem\_trans-NR , B:SBF\_like-NR, Aligned helices: A:1-5, B:1-5, E-value: 160

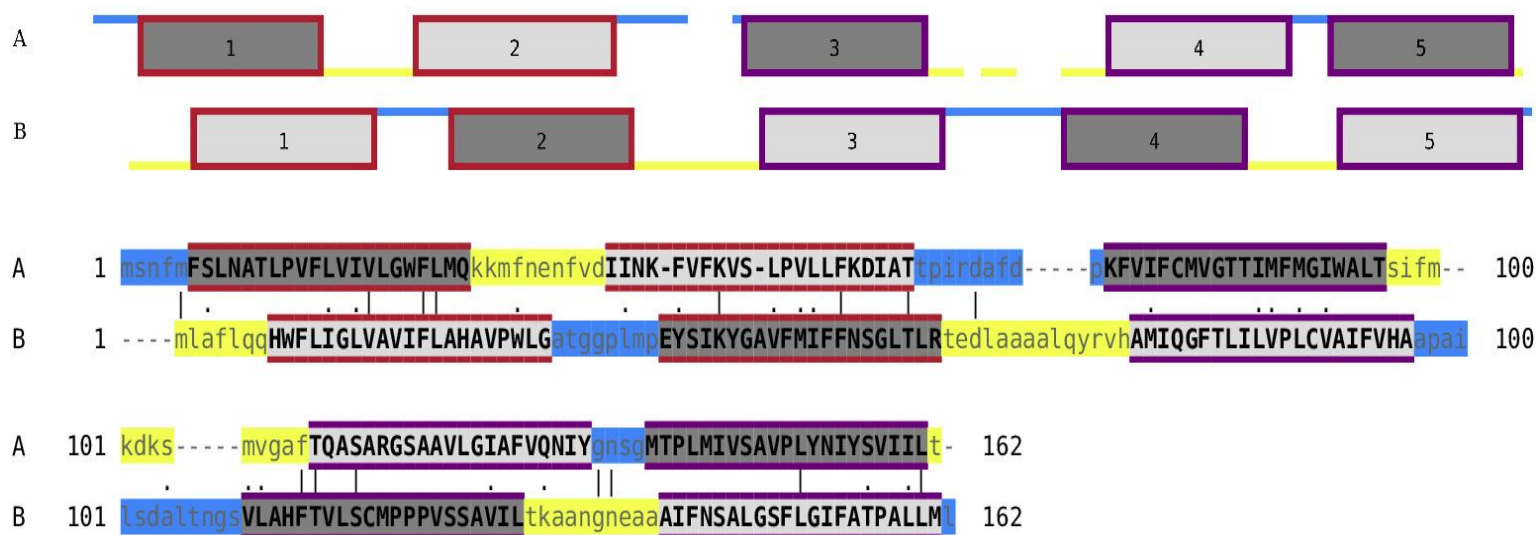

(b) SBF\_like

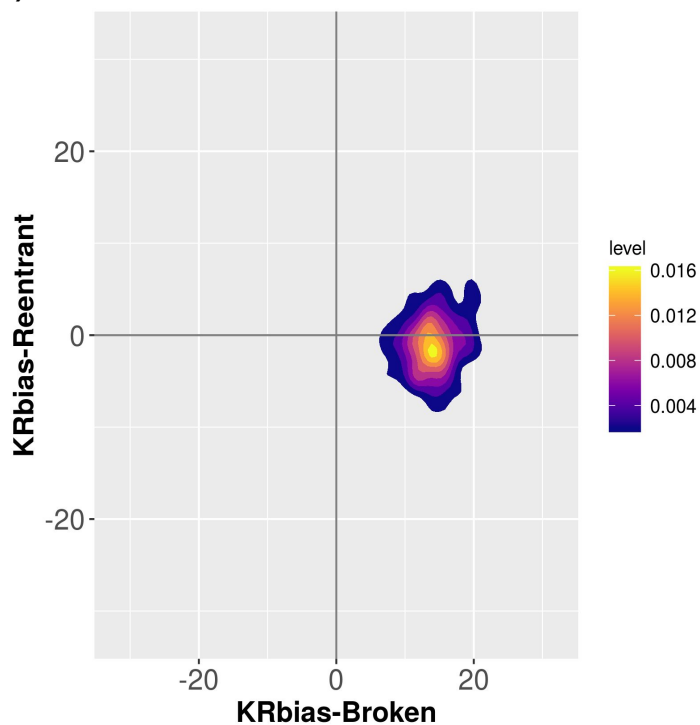

(c) Mem\_trans

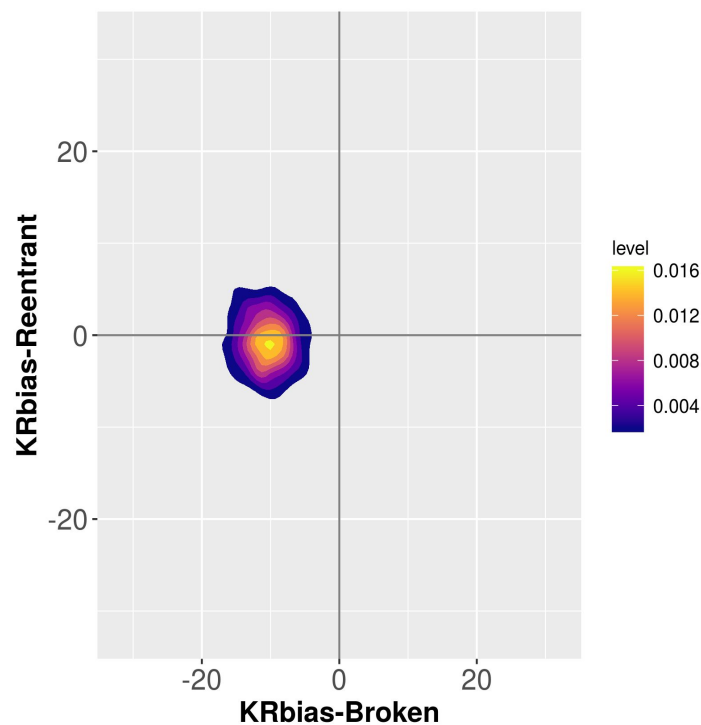

Supplement: S7 Fig — (a) Sequence and topology alignment between SBF_1 and Mem_trans N-terminal repeat units. (b) KR-bias plots showing the orientation of the SBF_like family. (c) KR-bias plots showing the orientation of the Mem_trans family. (PDF) [file pcbi.1009278.s007.pdf]

A: Na\_H\_Exchanger\_2, B: Na\_H\_Exchanger\_1, E-value: 3.5e-32

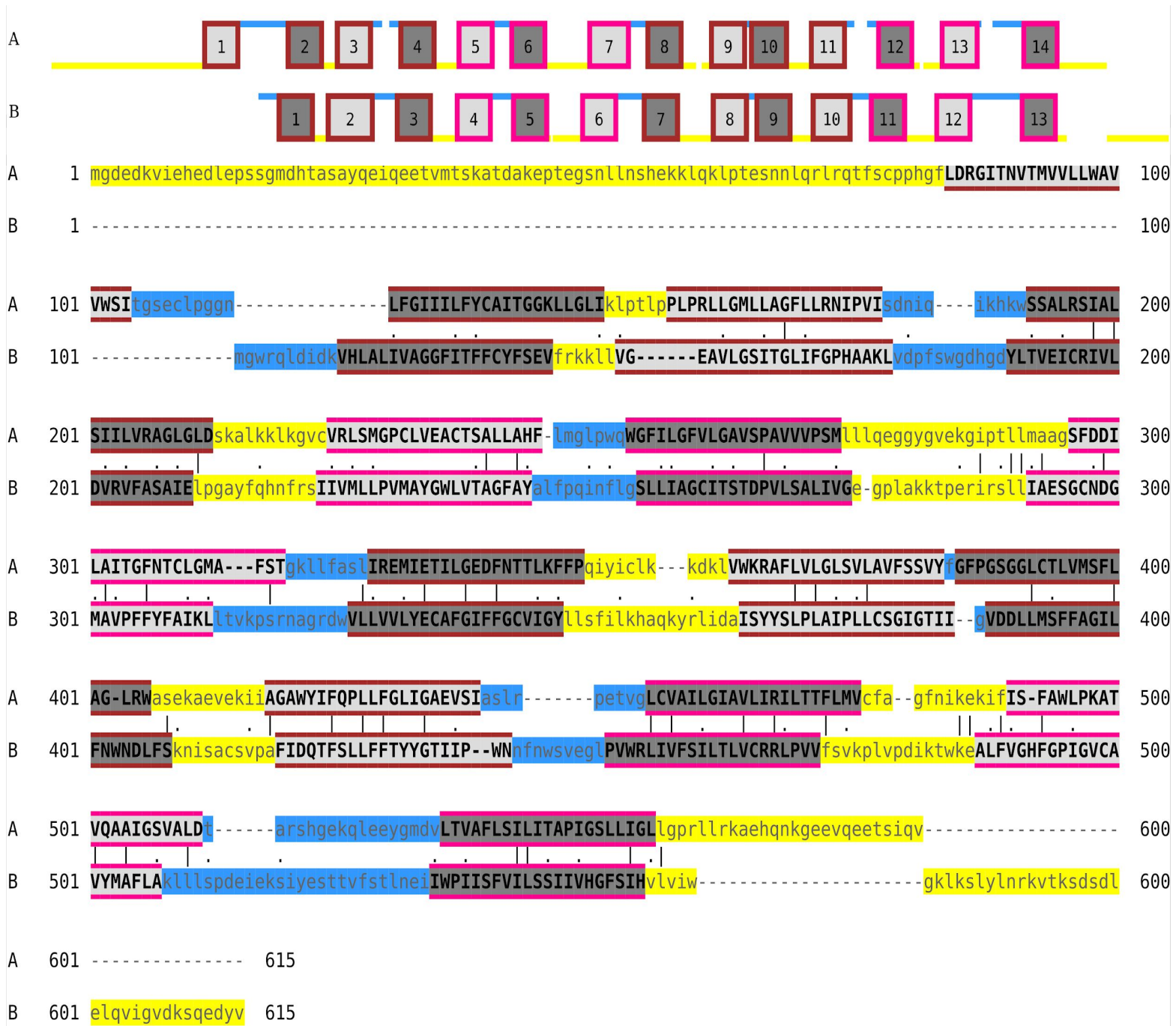

Supplement: S8 Fig — (PDF) [file pcbi.1009278.s008.pdf]

A: Glt\_symporter, B: 2HCT, E-value: 1.5e-30

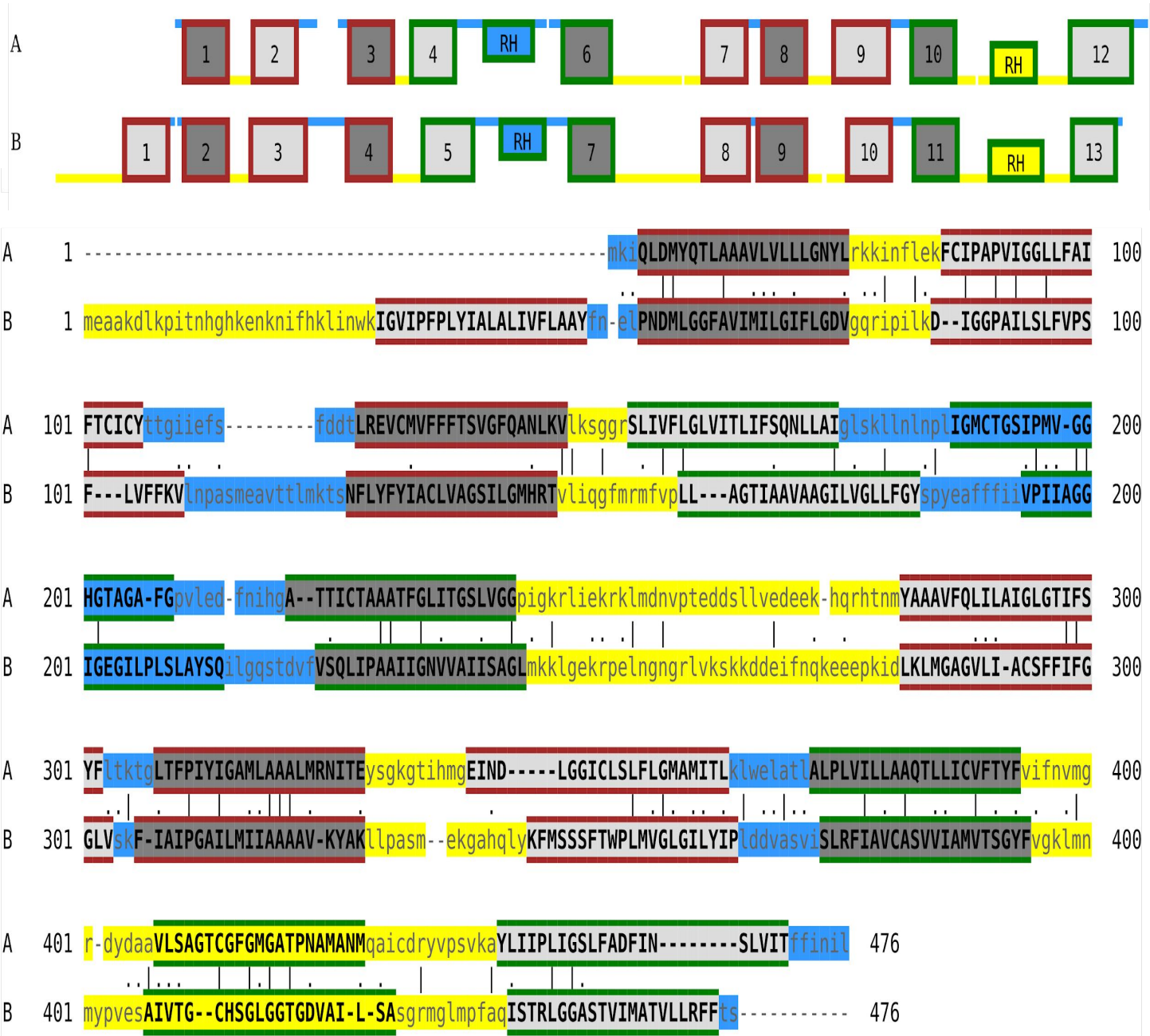

Supplement: S9 Fig — (PDF) [file pcbi.1009278.s009.pdf]

(a) A: Na\_H\_Exchanger\_1, B: KdgT ,E-value: 0.0011

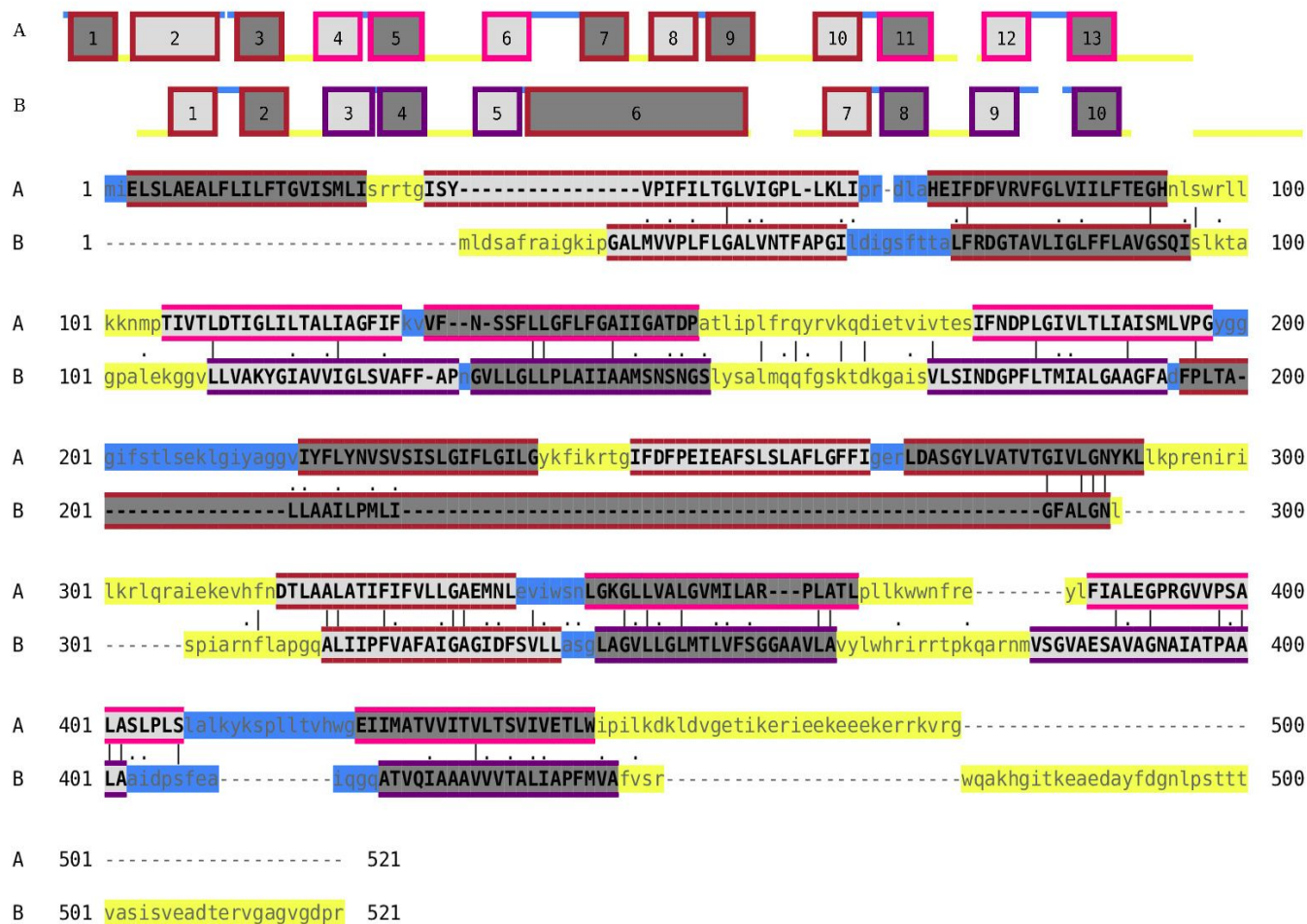

(b)

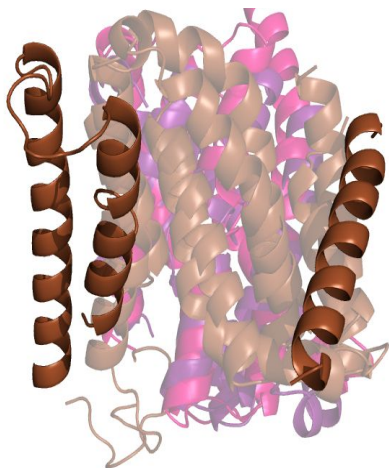

Supplement: S10 Fig — (a) Sequence and topology alignment between pairs of families from different fold-types containing full-length Na_H_Exchanger_1 and KdgT. (b)Structure superposition of families belonging to two families with known structure (PDB id: 4n7w, 4bwz). The gain of helices in one of the broken transporters is highlighted in brown. (PDF) [file pcbi.1009278.s010.pdf]
