# Supplementary material for: The evolutionary history of topological variations in the CPA/AT transporters
Source: PLoS Comput Biol. 2021 Aug 17;17(8):e1009278. doi: 10.1371/journal.pcbi.1009278 (PMC8396727; doi:10.1371/journal.pcbi.1009278)

(a) A: Na_H_Exchanger_1-NR, B: DUF819-CR, Aligned helices: A: 1-6, B:7-12, E-value: 0.061

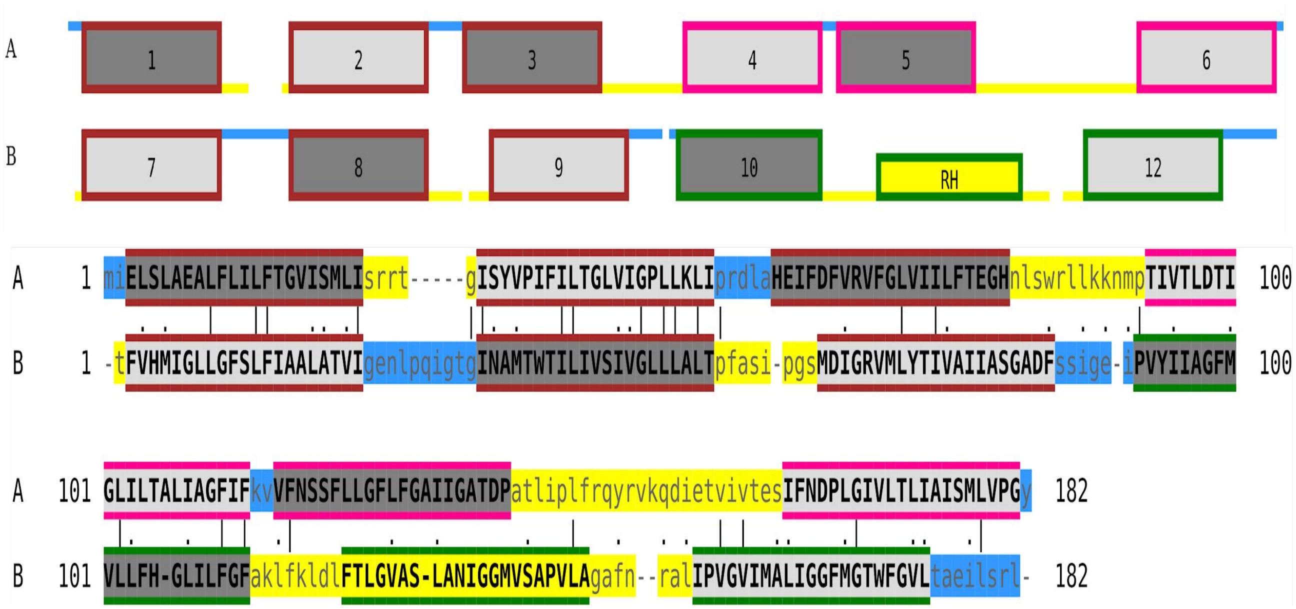

(b) A: Na_H_Exchanger_1-NR, B: DUF819-NR, Aligned helices: A:1-6, B:1-6, E-value: 1.7e-09

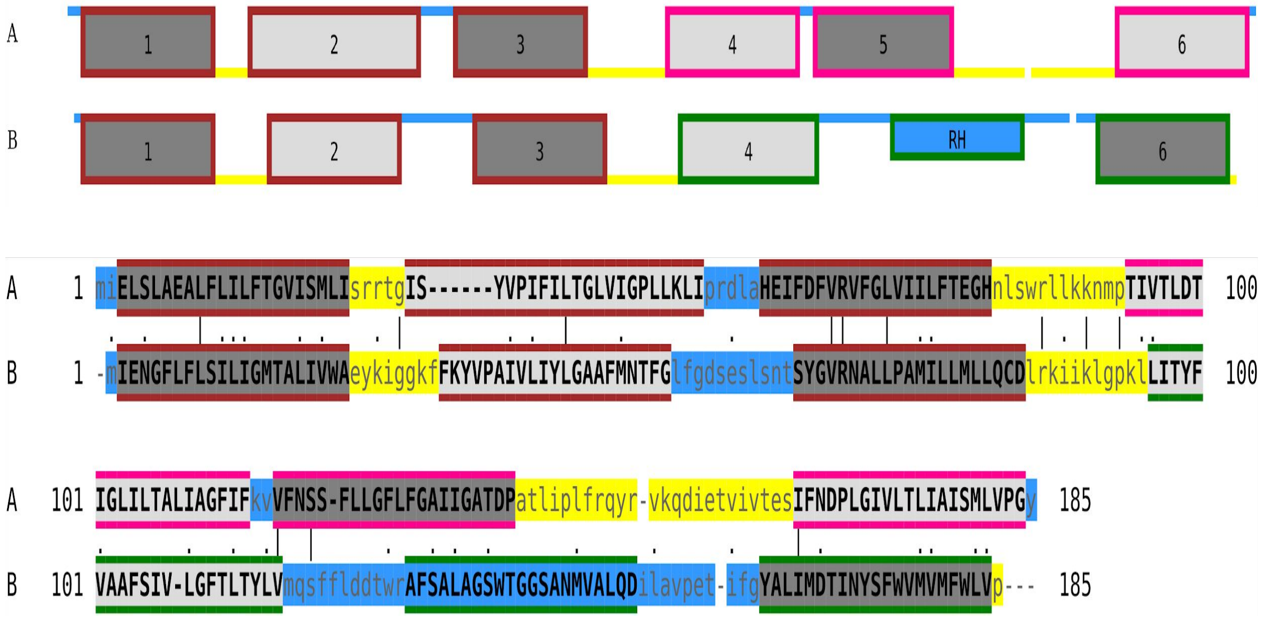

Supplement: S3 Fig — (a) Sequence and topology alignment between Na_H_Exchanger_1 N-terminal repeat and DUF819 C-terminal repeat (b) Sequence and topology alignment between Na_H_Exchanger_1 N-terminal repeat and DUF819 N-terminal repeat. (PDF) [file pcbi.1009278.s003.pdf]
