# Supplementary material for: The evolutionary history of topological variations in the CPA/AT transporters
Source: PLoS Comput Biol. 2021 Aug 17;17(8):e1009278. doi: 10.1371/journal.pcbi.1009278 (PMC8396727; doi:10.1371/journal.pcbi.1009278)

(a) A:AbrB-NR , B:Glt_symporter-CR, Aligned helices: A:1-5, B:7-11, E-value: 3e-05

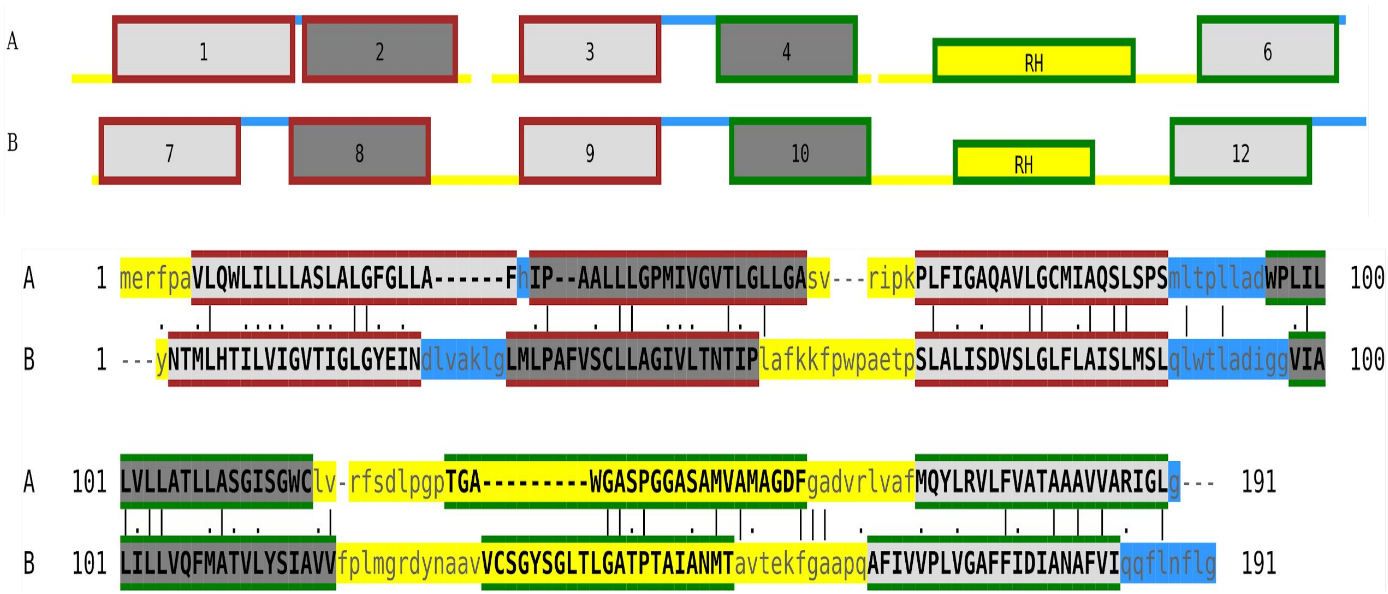

(b) A:AbrB-NR , B:Glt_symporter-NR, Aligned helices: A: 1-6, B:1-6, E-value: 6.4e-05

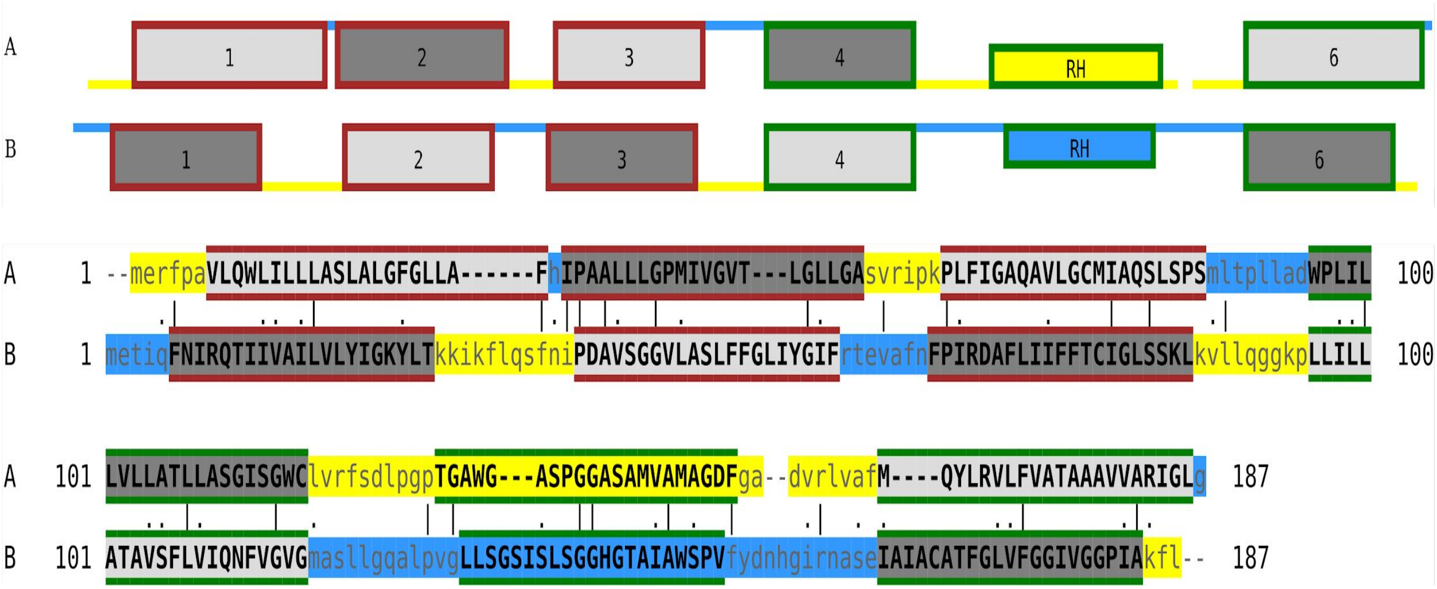

Supplement: S6 Fig — Sequence and topology alignments between Glt_symporter and AbrB families (a) Sequence and topology alignment between Glt_symporter N-terminal repeat and AbrB C-terminal repeat. (b) Sequence and topology alignment between Glt_symporter N-terminal repeat and AbrB N-terminal repeat. (PDF) [file pcbi.1009278.s006.pdf]
