# Supplementary material for: The evolutionary history of topological variations in the CPA/AT transporters
Source: PLoS Comput Biol. 2021 Aug 17;17(8):e1009278. doi: 10.1371/journal.pcbi.1009278 (PMC8396727; doi:10.1371/journal.pcbi.1009278)
Supplement: S1 Table — Meff score calculated for each family MSA, the Pcons-score, the TMscore for the proteins with resolved structure, and the ratio of contacts satisfied in the resulting model. The ratio of the satisfied contact was calculated for a number of contacts equal to the length of the proteins (L) considering the contacts distant more than 5 residues in the sequence. (PDF) [file pcbi.1009278.s011.pdf]

S1 Table: Information about the MSAs for each family. Meff score calculated for each family MSA, the Pcons-score, the TM score for the proteins with resolved structure, and the ratio of contacts satisfied in the resulting model. The ratio of the satisfied contact was calculated for a number of contacts equal to the length of the proteins (L) considering the contacts distant more than 5 residues in the sequence.

**Families belonging to CPA/AT transporters**	**Meff**	**Pcons**	**TMscore**	**Satisfied contact**
SBF_1	1055	0.62		0.77
SBF_2	1130	0.88	0.97	0.92
SBFlike	1133	0.81		0.91
KdgT	120	0.87		0.80
Mem_trans	1028	0.69		0.87
Sbt_1	192	0.56		0.81
Na_H_antiport_1	828	0.86	0.96	0.91
NA_H_Exchanger_1	1290	0.77	0.91	0.87
NA_H_Exchanger_2	90	0.72		0.76
Asp_Al_Ex	445	0.68		0.91
Glt_symporter	610	0.94		0.84
DUF819	575	0.93		0.83
AbrB	952	0.93		0.87

2HCT	151	0.68	0.91	0.74
OAD_beta	113	0.67	0.66	0.63
PSE_1	990	0.93		0.95
PSE_2	103	0.73		0.59
LysAB	175	0.52		0.66
LysA	598	0.96		0.31
LysB	278	0.94		0.81
LrgAB	227	0.52		0.72
LrgB	633	0.94		0.90
LrgA	1032	0.86		0.48
