# Supplementary material for: The evolutionary history of topological variations in the CPA/AT transporters
Source: PLoS Comput Biol. 2021 Aug 17;17(8):e1009278. doi: 10.1371/journal.pcbi.1009278 (PMC8396727; doi:10.1371/journal.pcbi.1009278)
Supplement: S2 Table — A structural template with the lowest E-value (in bold) is used to identify the broken or reentrant type. Additionally, identification of the type of transporters based on KR bias calculations is chosen based on the highest mean KR bias value (shown in bold). Two E-values are provided for the broken structure template of the Mem_trans family. Mem_trans does not align full length with the structure template. It instead aligns with the repeats in N- C-terminal way. (PDF) [file pcbi.1009278.s012.pdf]

S2 Table: Identification of Broken or reentrant type of transporter. A structural template with the lowest E-value (in bold) is used to identify the broken or reentrant type. Additionally, identification of the type of transporters based on KR bias calculations is chosen based on the highest mean KR bias value (shown in bold). Two E-values are provided for the broken structure template of the Mem_trans family. Mem_trans does not align full length with the structure template. It instead aligns with the repeats in N- C-terminal way.

**Family**	**Broken structure template**	**Reentrant structure template**	**Mean KRbias (Broken/ reentrant models)**
1.Na_H_antiport_1	Crystal structure **1ZCD (2.6E-62)**	5A1S(0.00046)	**18.47**/-3.10
2. Na_H_Exchanger_1	Crystal structure **4CZ8(2.1E-42)**	5A1S (5.3E-07)	**-14.73**/3.46
3. Na_H_Exchanger_2	**4CZB (8.4E-34)**	5A1S(5.1E-08)	**14.23**/-2.93
4. SBF_1	Crystal structure **4N7W(1.1E-44)**	5A1S(7.1)	**11.38**/1.63
5. SBF_2	**4N7W(3.3E-31)**	5A1S(5.2)	**-10.72**/0.72
6. SBFlike	**4N7W(2.4E-36)**	5A1S(0.39)	**14.04**/-0.99
7. KdgT	**3ZUY (4.9E-13)**	5A1S(0.033)	**12.72**/4.03
8. Mem_trans:	**4N7W (0.00012) (1.8E-16)**	5A1S(5.5)	**-10.71**/-0.31
9. 2HCT	4CZB (6E-05)	Crystal structure **5A1S (1.6E-59)**	-0.46/**14.67**
10. DUF819	4CZB (0.00068)	**5A1S(1.8E-30)**	3.94/**-11.21**
11. Glt_Symporter	4CZB(0.0005)	**5A1S(6.9E-28)**	3.39/**-13.68**
12. AbrB	3ZUY(0.027)	**5A1S(5E-6)**	3.36/**13.99**
13. Asp_Al_Ex	4CZB (0.00041)	**5A1S(4.5E-8)**	8.12/**-14.17**
14. PSE_1	5BZ3(0.15)	**5A1S(5.3E-13)**	1.78/**13.1**
15. PSE_2	4CZB(0.78)	**5A1S(8.6E-8)**	1.79/**9.76**
