# Supplementary material for: The evolutionary history of topological variations in the CPA/AT transporters
Source: PLoS Comput Biol. 2021 Aug 17;17(8):e1009278. doi: 10.1371/journal.pcbi.1009278 (PMC8396727; doi:10.1371/journal.pcbi.1009278)
Supplement: Attachment — Submitted filename: Reply-to-reviewers-PlosCB.docx [file pcbi.1009278.s013.docx]

Paper 1:

General Comments

1. The authors should do a better job convincing the reader why yet another classification of transporters is needed. Pointing out discrepancies between the current classifications is not enough since this new classification will bring yet another set of discrepancies.

The main goal of this paper is not to provide a completely novel classification. We actually think several classifications are quite good - but not complete. The TCDB classification is correct- but it misses the fact that CPA and BART families are related, which is pointed out in Pfam etc. Anyhow, we have now removed the section that compared various classification schemes. Now, we group transporter families belonging to the same fold-type based on their topology annotation and modelled structure only. Fold-types are defined structurally by similar repeat units found in a group of families. Fold-types are vital to this work as they provide a structural and evolutionary relationship of how the repeat unit evolves into different fold-types in the CPA/AT transporters. This basically extends the classifications Pfam, TCDB, OPM or CATH, by dividing the CPA superfamily into three distinct fold-types, broken, reentrant and RHR. Most likely these are evolutionarily separated into three groups but the evidence for monophyletic separation into three groups is not very strong, so it is possible that several transitions between these groups have occurred.

We have further replaced the names 5 helical broken, 7 helical broken and 6 helical reentrant fold-types with new and familiar names derived from the widely used TCDB database. Also, the addition of more families and merging of the contents of the second paper led to the inclusion of another fold-type namely, Reentrant-helix-reentrant fold-type. Families that belong to CPA/ BART superfamily in TCDB and/or acquiring a conserved 7, 6 and 5 helical conserved C-terminal repeat unit are classified into CPA-broken, CPA-reentrant and BART fold-types respectively. All families with Reentrant-helix-reentrant are assigned to Reentrant-helix-reentrant fold-type.

2. In the beginning of the results section it is explained that two new families were added, an explanation was provided alluding to the need of reliable alignment to other families, however it is not explained why. Conversely, two families were excluded because they are distantly related, how is threshold for distance defined? And why are those families present in the family in the first place?

We had used the Pfam clan (CPA_AT: CL0064) as a starting point to build our CPA/AT transporter dataset. Pfam clans are not always updated therefore we added the following missing families to our investigation of this clan:

(1) 2HCT family has not been classified into any Pfam family. But it is very clear from our HHsearch alignments that 2HCT is evolutionarily related to other families of CPA/AT transporters (Figure 3)

(2) The AbrB family has been misclassified into the Mem_trans clan in Pfam. HHsearch alignments of AbrB family versus all Pfam families from our analysis clearly shows that this family is evolutionarily related to families from Pfam’s CPA clan (Figure 3). It does not detect any family from the Mem_trans clan as being evolutionarily related. We also clearly observe that both 2HCT and AbrB belong to the CPA-reentrant-fold-type based on the conserved 6 helical reentrant C terminal repeat unit. This is the rationale for including both the families in CPA/AT transporter dataset.

All the full length and repeat level alignments of 2HCT with other families and AbrB with other families are provided in CPAfold database. The e-value of the alignments are also provided.

In our previous version, we had excluded 4 Pfam families namely, OAD_beta, (PF03977) lys_export (PF03956), sbt_1 (PF05982) and *LrgB* (PF04172). These are now included. However, figure 3 clearly shows that all 4 above listed families are distantly related to the other families (OAD is, in particular, a clear outlier) and makes very few connections with other families. By including these distant families in our current version, we have assigned fold-types and discussed their fold-evolution. We thank the reviewers for this useful suggestion.

3. Figure 2, deals with the DUF819 family. I would suggest to incorporate this into the actual figure to make it clear for the reader when looking at the figure before reading the captions

We agree that it brings in more clarity by incorporating the family name in the figure. We have now made this change in the revised manuscript. Pls refer to Figure 9.

4. The KR-bias is used in Fig. 2 before it is properly introduced in the manuscript. I suggest you include a brief definition in the figure caption to correct this continuity issue.

We have now introduced KRbias in the figure caption. We have mentioned how KR-bias calculation has been carried out for the DUF819 family to correctly predict if it is a broken or reentrant topology. The continuity issue has now been resolved in the revised manuscript.

5. The MSA-MSA pairwise alignment is not clearly defined. Please include a brief explanation in the fold types section.

A family MSA was generated for each family. The representative sequence of each family was searched against Uniclust30 using the HHblits program with default settings generating a family MSA. HHsearch program searches the query family MSA against the MSA of all the Pfam families of Pfam-A_v32. The output is the query family MSA and templates Pfam family MSA. These query-template MSA are referred to as MSA-MSA alignment in this work. We have now included this explanation in the revised manuscript.

6. The manuscript uses the concept of the “positive inside rule” as a ground truth, is this assumption extensive and general? As a non-expert I would doubt this always has to be true, could the authors clarify ?

The “positive-inside rule” is the preferential occurrence of positively charged residues (lysine and arginine) at the cytoplasmic loops of transmembrane helices. A large-scale study with statistical observations of 107 organisms belonging to Eubacteria, Archaea and Eukarya containing membrane proteins strongly support positive-inside rule. This rule exists for most if not all organisms. In general, KR bias can be applied to any genome for topology prediction. We have now emphasized the ubiquitous occurrence of positive inside rule in membrane proteins in the manuscript. We have cited the papers related to the extensive and general role of positive-inside rule.

7. In step 1 of topology annotations, the authors use the reference proteome sequences for Pfam. Typically this set is much smaller than those including all the Uniprot proteins. What is the rationale to use only the reference proteomes and not all available sequences?

The reference proteomes are complete proteomes that represent taxonomic diversity. In particular, they include the well-studied model organisms that are of biomedical and biotechnological interest. It is very important that seed MSA represents the full-length sequence. This is important to get the complete topology. The prevalence of fragmented sequences and partially sequenced genomes can lead to the assignment of incomplete topology or wrong topology and error. We were careful to include only the complete genomes to get accurate topology predictions. Smaller size did not matter for topology predictions and topology annotation of the family. We have now included the rationale of using only reference proteomes for the topology annotation in the revised version.

8. Please include a section describing possible applications or use cases of this database. For example Pfam can be used as input MSAs for contact prediction, what about this database?

The CPAfold database can be useful to experimentalists interested in studying the structure, function, mutation and design of any protein belonging to this fold.

1. 3D structure models are available for all the families in this fold. Functional and mutational studies can be supported by these models.
2. Novel reentrant motifs for the reentrant type transporters have been identified.
3. Full length and repeat level topology and sequence alignments of a particular protein family with all the other families in the fold are provided. This becomes helpful for comparison of structure and function of related families of interest. The alignments are very easy to follow figures with topology being mapped on to the sequence alignment.
4. Users can check which family his/her protein of interest belongs to. The protein of interest belonging to CPA/AT transporters can be aligned with the family MSA belonging to all the families using the HHalign program. The protein can be assigned to that particular family that shows the lowest e-value with protein of interest.
5. Topology and UniProt id of the representative sequence for each family are available. Topology seed MSA can be used to extrapolate the topology, repeat regions, core and scaffold regions for the protein of interest. This can be carried out by aligning the protein of interest and topology seed MSA by the HHalign program.
6. Once a family has been assigned to the protein of interest, fold-type, broken/reentrant core domain can be automatically assigned.
7. We also have the mapping of classification with other databases such as OPM, Pfam, CATH, TCDB. After the family has been assigned to the protein of interest, it is possible to look at the information available from other databases to explore its function and evolution.

Minor details

1) Abstract. Replace “losses of core helices” with “loss of core helices” : Done

2) Author summary. Line 60, add a period between “superfamily” and “We”: Done

3) Page 5, line 101. Replace “repeat (In ..” With “repeat (in ..”: Done

4)Page 6, line 115 , replace “enter and exit” with “enters and exits”:Done

5) In table one some cells start with a semicolon (;) what is the reason for this?

The words before the semicolon refer to the name of the clan/Superfamily from different databases. The words following the semicolon refer to the family being discussed in that row. Some families are not assigned to any superfamily. In that case, there is no word preceding the semicolon. For example, **;** 2HCT (PF03390). 2HCT Pfam family or PF03390 is not assigned to any Pfam clan. There is nothing preceding the semicolon. We have now explained this in the table 1 legend to avoid confusion.

6) Fig. 3 captions, line 193, replace “is represented” with “are represented”: Done

7) Page 12, line 210, replace “helices themself” with “helices themselves” or remove “themself”: Done

8) Page 14, line 238. I suggest to remove “Anyhow” at the beginning of the paragraph: Done

9) Page 17, line 281. Replace “family have” with “family has”: Done

10) Page 21, line 374. Replace the comma with a period in “selected, In case”: Done

11) Could the authors explain what is the PDBmmCIF70_22_May database?

This is the database that belongs to the HHsuite program. For each protein chain in the PDB, multiple alignments with iterated HHblits searches over Uniprot20 is built. The alignments are transformed into HMMs. The query HMM is compared to the database of HMM-based on PDB chains to generate the query-template alignments. PDB_mmCIF70 are filtered with representative sequences with a maximum of 70% sequence identity selected. We have explained the generation of the HHsuite database in the revised manuscript.

Reviewer #2:

In the submitted manuscript authors present sequence- and structure-based classification of the CPA/AT transporters superfamily. Such a classification is an important step towards understanding the evolution of these proteins, however, experiments being at the base of this work may lack robustness.

- Fig. 2. “The figure shows a reordered topology alignment” – what does reordered mean in this context? What is the meaning of helix numbering (also in Figs. 4, 5, and 6). Is the presence of the reentrant helix (RH) in N or C repeat mutually exclusive? These issues should be addressed in the introduction. Also, figures’ legends would benefit from clarifications. Now it is hard to understand them without referring to the methods.

We obtain a seed MSA for each family. This MSA is used to generate a phylogenetic tree using the Fasttree program. The MSA is now reordered according to the branching order from the phylogenetic tree. We name this as the reordered sequence alignment. We carry out topology predictions for all the sequences in the seed MSA. The topology is mapped on to the reordered MSA to obtain a reordered topology alignment. The whole objective of reordered topology alignment is to observe clusters of sequences with conserved topology. Very closely related proteins within a cluster would have the same topology. We have cases where there are two evolutionarily distinct clusters with different topology after the reordering based on the phylogenetic tree. Then the families with different topologies are split into two subfamilies. The second use of reordered topology alignment is to weed out the topology errors arising from topology predictions. Proteins with different topologies not seen as a cluster are considered to be errors of topology prediction. Thus the reordered topology alignment is important to annotate topologies for the protein family and to obtain the initial topology of the annotation pipeline. We have now tried to explain this better both in the figure and in the manuscript.

A reentrant helix in the N-terminal repeat is always followed by another reentrant helix in the C-terminal repeat. This is also true for a broken helix. This is required for the ions to bind near these non-canonical helices and bring about transport. A reentrant helix in one repeat cannot coexist with a broken helix in the other repeat unit or vice versa. This statement has now been explained in the introduction section of the revised manuscript.

- It is unclear to me how Fig. 3 was used to define the three groups. First, the title of the figure is “Repeat units are clustered based on conserved C-terminal repeat” (also Pg. 19, Ln. 320) but it shows 30 units which correspond (I guess) to N and C terminal repeats of the 15 families from Table 1 (this is also stated on page 10, lines 182-183). It is not clear when the authors refer to a single (N or C) repeat or the full fusion protein. Second, I don’t understand how the investigation of the dendrogram could have led to the definition of the groups. The purple group actually comprises two sub-groups that share no similarity, the same is for the green and pink groups (I don’t see how Fig 3 could support their existence – page 11, lines 196-197). I suggest representing the all-vs-all similarities in a form of a cluster map (e.g., with CLANS [T Frickey 2004] or a similar tool) and using such a representation for defining groups. Currently, it looks like the manually-defined groups were mapped onto the dendrogram just to state that they were actually obtained from it.

Thanks a lot for your useful suggestions. We have now grouped transporter families belonging to the same fold-type based on their topology annotation and modelled structure only. Fold-types are structurally similar repeat units found in a group of families. Based on your suggestion, we have made a network based on full fusion protein and another network at the level of repeat units (see Fig 4). These fold-type assignments are then mapped on to the network generated.

We agree that both the dendrogram and network do not clearly cluster based on fold-types. We accept that the fold-types need not necessarily be evolutionarily closely related always. It is possible that some transitions between fold-types happened more than once, there is not enough data to support or reject that. But, we do anyhow have some important learnings from our fold assignment and network analysis. We have inferred important evolutionary relationships across fold-types. We also observe various evolutionary events such as duplications, broken-reentrant helix transition, reentrant helix-loop transitions, shuffling of repeat units etc, which we believe are important to analyse to understand the evolution of this group of families better. We have now discussed in detail the findings from our network analysis in detail in the manuscript. We have included all the families based on your suggestion.

We initially tried to use the CLANS program. However, we found that all versus all blast comparisons available in CLANS was not suitable due to extensive sequence divergence within protein families within the fold. As an alternative, we opted to use the scores from HHsearch of pairwise HMM-HMM alignments The weighted network based on scores were obtained from a python library “NetworkX” and are now presented in Fig 4.

- It is not clear what Fig. S6 actually shows. Please clarify. Pg. 12, Ln. 212-214. Why helix gain/loss would be restricted only to the C-termini scaffold subdomain? Can’t the broken to reentrant transition occur in the N-termini core domain? Pg. 14, Ln. 242-250 – why only one example was given? Are there other examples that would support different scenarios (for example, a transition that does not involve duplication of a single repeat but rather a transformation of a two-repeat fusion protein)?

We have now removed Fig S6 and text associated with it in the merged version of the paper. This is because the histogram was only used to show the nature of the dataset. We have also shown the strength of the evolutionary relationship between family pairs (log10 e-value) that show different types of topology variations.

This is an explanation for the broken-reentrant transition. It should be noted that the formation of inverted repeats is important for the function of the CPA/AT transporters. Let's consider the example of 13 helices long Na_H_exchanger_1 family (Broken transporter) and 12 helices long DUF819 (Reentrant transporter). Since DUF819 has reentrant helices, it needs to be 12 helices long. This helps it to provide the inverted nature. The broken transporter on the other hand needs to have the extra helix in the N terminal of the C-repeat in order to achieve the inverted nature of repeats. Any broken-reentrant pair of transporters shows the same trend. Gain or losses in the N terminal repeats might or might not occur. This does not affect the broken-reentrant transition.

We report the comparison between the N-repeat of DUF819 (Reentrant) and the N-repeat of Na_H_Exchanger_1 due to their lowest E-value. N-repeat of Na_H_exchanger_1 is also related to N-repeat of 2HCT (reentrant) but has a higher E-value than the former (Thin edges in the repeat level network) and Glt_symporter (Fused full protein network). We wouldn’t tell that the evolution of all the families has occurred at a repeat level. Most of the evolution has occurred as a fused full protein. For example, it is clear from both the repeat and full-length network that evolution within the fold-types occurs at full length. But, the initial transition between the broken and reentrant transporters occurred by duplication of the repeat. (All the alignments are available in the CPAfold database). We have now rewritten these portions that were not clear in the current version.

- In general, the presented evolutionary scenarios (Pg. 12-17) are speculative and are based on anecdotical observations rather than the investigation of all the available data (comparison of the repeating units). In my opinion, the work would benefit from having a figure in the main text (maybe a schematic tree?) that would summarize all the families, including the relative similarities between their repeats, their features (core helix type, orientation, etc.), and list the possible scenarios of duplications/fusions/swaps, etc. S16 Fig. may be a good starting point for such a figure.

We have inferred the evolutionary scenarios from the investigations of HMM-HMM alignments between families in the CPA/AT transporters using HHsearch program. The scores of these pairwise relationships are shown in the form of a repeat network. The other analysis which supports these evolutionary transitions are listed below:

(1) Loop-reentrant transitions: Structural comparison of the crystal structure of 2HCT (CPA-reentrant) and PSE_1 (Reentrant-helix-reentrant)
(2) Broken-reentrant helical transitions: Reentrant motifs, Role of hydrophobicity, KRbias and packing of helices.
(3) Internal duplications: MSA-MSA alignments between N and C repeat, Reentrant motifs.
(4) Shuffling of repeats: MSA-MSA alignments between all combinations of N- and C-repeats from SBF and Mem_trans families.

We had to shift a lot of details to supplementary due to space constraints. However, we have now included a summary figure with all the details in it.

- It is not clear why the four families were excluded from the analyses.

In our previous version, we had excluded 4 Pfam families namely, OAD_beta, (PF03977) lys_export (PF03956), sbt_1 (PF05982) and *LrgB* (PF04172). This was because these families were distant and made very few connections with other families. We had analysed from the perspective of the superfamily (Figure 4a & 4b). We have now included all these families in the revised version. By incorporating all the families, we provide some insights into the evolution of fold-types in the CPA-fold.

- The authors do not comment on the fact that in some cases the two repeats are not in a single protein chain but are rather encoded separately.

In our previous version, all the protein families that were discussed had both the repeats encoded in a single protein chain. In our updated manuscript, we include more families. Some of these newly added families have two repeats that are not in a single protein chain.

(1) LrgAB operon includes two different proteins LrgA and LrgB. LrgA and LrgB interact together to form a functional dimer.
(2) In another case, few reference proteomes show the coexistence of LysA and LysB. This shows that both these proteins encoded by different genes interact with each other to form a functional protein.

Details of both these families are provided in the CPAfold database.

Reviewer #3: The work described by Sudha and colleagues, describes the effort to use a number of sequence and structural bioinformatics tools, to study the evolutionary history of the CPA/AT family.

Clearly this work, is relevant for a very specialized crowd that studies the CPA/AT superfamily and I have a hard time seeing the impact of this work outside of that community. On a more positive note, the authors did make the effort to summarize their findings on a searchable database that can serve the community that studies these proteins to use the findings of this paper.

We do accept that the paper focuses on a particular group of transporters. However, the evolution of different types of fold-types, structural variations, evolutionary events will hold true to all transporters containing repeat units. Also, the methodology adopted to infer topology, structures and fold-types can be extended to other transporter clans from Pfam. So, this work is relevant to people interested to understand transporter protein structure and evolution. We have now explained this in the conclusions section of the revised manuscript. Thanks for appreciating our idea of presenting the findings of this work in a database.

Another point that strikes me in the manuscript is related to the proposed evolutionary pathways as far as my understanding goes these evolutionary pathways are just “putative solutions” and sometimes the language in the manuscript oscillates in between this is a proposed mechanism and this is the only event possible. Would be important to be consistent in this section.

We have now clearly explained how the results from our work support all the evolutionary scenarios.

Specific comments

CPA/AT family in the title is generally not a great way to present the work to a broader audience as many scientists will not know what the CPA/AT superfamily is

Our work is useful in general to understand transmembrane protein fold evolution, types of structural variations, different evolutionary events that bring about these variations. We have also discussed a method to predict the topology, fold-type and 3D structure using our integrated method. It can be extended to all the other repeat-containing transporter clans in Pfam. However, we have carried out the analysis only for the CPA/AT transporters. So, we included the name of the transporter in the title.

- line 60 missing a punctuation

It has been corrected.

-line 63 would or could ?

Changed to Would

- in terms of notation I find figure 1 c confusing as clearly we do not have 2 and 3 helices – but rather 4 and 6

We agree that it is confusing. We meant that each subdomain has 2 helices. Both the N- and C-terminal scaffold subdomains are shown. So in total, there are 4 helices. We have now made this clear in Figure 1 of the revised manuscript.

######################### PAPER 2 #########################

Reviewer #1: see attachment

Reviewer #2: In the present submission “Identification of a novel fold type in CPA/AT transporters by ab-initio structure prediction” C. Bassot et al. investigate the large class of CPA/AT transporters via molecular modeling and contacts prediction tools to gain novel insight into the structure of CPA/AT subfamilies. They split the entire superfamily into distinct individual families with different structural features, namely the number of transmembrane regions. This structural heterogeneity makes the straightforward use of homologue modeling tools challenging, as these tools rely on structural closeness. In the present submission, the authors manage to use ab-initio modeling to address this challenge and carefully validate their method on known structures before predicting the unknown folds.

Summed up, CPA/AT transporters are ubiquitous membrane transporters and a topic of high biological interest. The present submission itself is well-written and investigates this class of transporters in high detail. The technical aspects are only very shortly described but a complementary web-server with complete data and a remarkable intuitive interface is present as is a git. There are only few parts which needs some work and should be addressed before publication (see below). After minor revision, the submission is suitable for publication.

Major Issues:

-

Minor issues:

-The technical discussion on “Generation of protein models and quality assessment” is very short and should be expanded.

Thank you for the comment. We expanded the section giving more detail about the trRosetta model generation

-The introduction is very focused on the biology of CPA/AT transporters. The introduction into structure prediction/ used methods is basically not present. For examples, while DCA is listed as a keyword, is nowhere mentioned in the manuscript; co-evolutionary analysis for contact prediction has strongly advanced influenced biomolecular structure prediction by harvesting the ongoing growth of sequential databases and providing information about spatial adjacencies by analyzing mutational pattern. The discussion on structure prediction/ co-evolution and how it has been used should be added.

Thank you for the comment. We have added a description of the contact prediction method in the Introduction.

-Figs 3a, b, c have very small labels on the axis

In the new version of the paper we removed the figure.

-Figs 4 ab have very small labels

In the new version of the paper we removed the figure.

-Some minor typos (e.g. in some versions of the title "CPA/AT transportersby" (missing space, possible copy & paste error?))

Thank you for the careful revision we revised the text.

############################################################################

Reviewer 2

The paper explores a diverse transporter superfamily. The paper expands knowledge about this group utilising both homology and contact assisted modelling showing the presence in some families of reentrant loops packed to form a fold that has not previously been documented. The authors also provide an evolutionary hypothesis for the formation of this reentrant feature.

Major comments

- The two Abstracts differ??
- In Abstract 2 it is durely incorrect to call a reentrant helix as transmembrane – it doesn’t cross the membrane

Thank you for the comment. We have now removed the adjective “transmembrane” when describing the noncanonical helices.

- Structure figures are not very useful
  - p.6 “Structurally, the core of the PSE_1 and 2HCT models are aligned, see Figure 1C” Fig 1C is completely uninterpretable. Maybe use ribbon rather than cartoon, deemphasise unimportant parts of the structure, present in stereo, use depth cueing… *somehow in fig 2.* Maybe deemphasise residues not aligned by TMsore, show in stereo, ribbon not cartoon etc

Thank you for the comment. We have now modified the figure according to the reviewer’s suggestion.

- writing far too small on Fig 3a

In the new version of the paper we removed the figure.

- Fig 5 legend is incomplete eg white grey TM helices. Why is the particular TM helix picked out in green? There is no a or b in the fig

Thank you for the comment. We have added the letters in the figure and extended the caption.

- p.17 The structure alignment tool of PyMOL is unpublished so its used should be discouraged. Why were two different structural alignment tools used? Which was used when? The built-in PyMOL tool works on the basis of an initial sequence alignment and therefore struggles to align distantly related proteins – it seems particularly inappropriate for work like this. More obvious alternatives like DALI or GESAMT would give more useful scores.

Thank you for the suggestion we repeated the paper analysis using TMalign instead of PyMol. The data didn’t change.

- p.18 It is unclear how the structure-based dot-plots are derived. We are told “In the structural alignment the shade of black corresponds to the distance between the aligned residues expressed in Ångstrom”. Which of the structure superposition tools was used? If the black-grey represent aligned residues how can some positions in one protein have multiple marks vs positions in the other protein? Are these figures in fact all-vs-all distance matrices? We assume these are the trRosetta models?
- The structures were superimposed using TMalign. The figure is in fact an all-vs-all distance matrix between the trRosetta models and in the case of figure 1 with 2HCT pdb structure 5a1s. We update the captions to better explain the figure.

Comments on cpafold website. Well-presented but

- Where are PSE_1 and _2?
- They have both been added correctly and shows up on the website
- Models are presented as CONFOLD Homology model and Tr Homology model (I assume trRosetta). Neither of these is a homology modelling protocol
- Models are now presented as either “Crystal structure” or “trRosetta model” to reflect the two types we are now presenting
- Taking <https://cpafold.bioinfo.se/details/Glt_symporter/> as a random example, the colours don’t always match the bilayer. Is that right? C-scaff 9 illustrates drawing non-helical structure as a helix
- The structures/models has been inserted into a bilayer using the PPM-server of the OPM-database. This means there can be discrepancies between the helices and the bilayers(the helices with a higher probability of being correct).
- Are the data in database deposited elsewhere too for more sustainable/guaranteed future access?
- Figshare synced against the github-repo for all data.

Minor comments and English issues

*Author summary*

“CPA/AT superfamily transporters …”

“non-canonical transmembrane helices: we named it reentrant-helix-reentrant” but whole sentence unclear

*Intro*

Replace PFAM with Pfam for consistency eg “LrgAB(in **Pfam** in the LrgB family)”

“In that paper we extended…”

Line “These newly added Pfam families are also evolutionarily related to the superfamily.” Seems redundant – why else would families be added other than an evolutionary relationship?

“According to our definition, the superfamily…”

“Na^+^-transporting”

“However, the middle helix has a non-canonical structure that can be either “broken” or “reentrant” which and is less hydrophobic than the other transmembrane helices.”

“Structurally the broken and reentrant helices form a similar, but topologically different, three-dimensional structure [5]“ Not clear exactly what is meant by structural similarity here. I would suggest a figure.

I don’t think the call to Fig 1B at the end of the Intro is helpful. We have no real idea what we’re looking at at this point.

p.3 Not sure what ‘(Ex1, Ex2, etc.)’ means here.

p.4 please put TM-scores in Table S2

p.4 ‘cum *grano salis’ is needlessly obscure*

*p.4 Why is the e-value rather than the p-value shown for HHpred results?*

*p.4 this sentence seems to be key for what follows but it is not made explicit what the structural difference is “*The C-terminal core domain in PSE is structurally different from all known structures in the CPA/AT superfamily.”

p.4 “…there was structural dissimilarity…”

p.5 Table 1. The e-values are very high but is it being argued that the matches are meaningful because the homology models align well to the trRosetta models? If so, state that clearly and put the TM-scores in Table 1.

p.5 RH is an odd abbreviation for reentrant region

p.6 “In particular, the homology model lacks the second reentrant helix” Which homology model, both? Do the predicted contact maps have features for the reentrant loop(s)?

p.6 *E. coli* not E.Coli

p.8 14.5% not 14,5%. How many aligned positions?

p.8 replace “can also note the internal symmetry” with “reveals an internal symmetry” (if I have understood this right)

p.10 ‘aligns well’ -> how well? Please quantify

p.12 why would the bit score be artefactually high for similar length proteins?

p.16 “that drastic topology changes can occur in membrane proteins.”

p.16 Neff values for the MSAs of proteins modelled ab initio would be of interest

p.17 20 trRosetta models were made yet the rest of the ms talks about ‘the’ trRosetta model. Which of the 20 was chosen? Why were more homology models not made similarly?

p.17 satisfaction of long-range predicted contacts could be used for orthogonal validation of ab initio models.

p.18 what “were calculated using the bit scores obtained by HHalign, see Figure 7a.”

We thank the reviewer for the careful revision. The majority of the comments where accepted or the sentence was removed/rewrite in the context of the merging of the two papers. The few we discuss are listed below:

*p.4 Why is the e-value rather than the p-value shown for HHpred results?*

In our opinion the e-value is clearer than the p-value and is the score and showed as well in the webserver version of HHpred

p.5 RH is an odd abbreviation for reentrant region

The abbreviation stands for Reentrant Helix we took at it as the most straightforward abbreviation.

p.17 20 trRosetta models were made yet the rest of the ms talks about ‘the’ trRosetta model. Which of the 20 was chosen? Why were more homology models not made similarly?

We chose the model with the highest pcons score. With the homology models this approach would not be possible because Swissmodel produced a single model per each template.

Reviewer #3: The submitted manuscript describes a study that appears to be the continuation of the work submitted by the same authors at the same time (PCOMPBIOL-D-20-02296). The study itself focuses on modelling the 3D structures of CPA/AT transporters with the aid of homology modelling and ab initio modelling tools. The main finding is the identification of potentially novel architecture among CPA/AT transporters.

Comments:

Pg. 4, Ln. 76-78. Did the authors exclude these benchmark structures from the Rosetta fragment library? If not, such a benchmark not meaningful. “a linear relationship between Pcons- and TM-score is evident, Figure S1” – this statement is way exaggerated considering the number of points.

We modified the sentence with there is “a correlation between…”. The benchmark structures are not part of the fragment library.

Pg. 4, Ln. 88-95. In few cases, the two modelling approaches yielded substantially different models. Among them, there was PSE which contains a novel RHR motif. Do I understand correctly that if there were no discrepancy between the modelling tools, the authors would have never spotted the peculiarity of PSE?

It’s correct. Using only the homology model, we will not be able to spot the novel fold.

Pg. 5, Ln. 109. “As described above the two models show substantial differences to the corresponding homology model(s)”. The two Rosetta models of PSE_1 and PSE_2? Fig 1b; is this the Rosetta model? Please clearly indicate in the text which of the two models (homology or Rosetta) is being referred to.

Thanks for the specified text.

Pg. 8, Ln. 134-143. Fig. 2 lacks the sequence comparison between LrgB and PSE. Please indicate that (Rosetta?) models are being compared. Not sure why the failed attempt of LrgAB modelling is mentioned (all conclusions are based on the modeling of the individual components)?

We remove this part in this version of the paper.

Table 1. I am not sure whether homology modelling on templates with E-value>1 is reasonable (the templates may be correct but the alignment, essential for the modelling, may be not). I suggest checking various HHpred parameters such as MAC-realignment and ensuring that the resulting alignments are consistent.

We are aware of the homology models. E-value > 1 is not reliable. We have removed these models because they were misleading.

Fig. 3. The captions are barely visible on a printout. Please make them larger.

In the new version of the paper, we removed the figure.

Pg. 10, Ln. 157. Shouldn’t be Fig. 3a not Fig. 3b?

Yes. Thank you for highlighting the mistake.

Pg. 14, Ln. 196-197. Figure 4 suggests that the RHR fold evolved independently in PSE, Lrg, and Lys. I suggest clustering all the families (individual repeats) to check whether PSE, Lrg, and Lys will end up together; if this is true then one could speculate about the common (non-convergent) origin.

Thank you for the comment. We performed a broader cluster analysis (Figure3) and from this analysis, it is quite clear that PSE evolved from (an ancestral) 2HCT. However, the origin of Lrg and Lys is not clear, it could be derived from the same origin, but it might also be independent. We have tried to clarify this in the paper.
