# Supplementary material for: The evolutionary history of topological variations in the CPA/AT transporters
Source: PLoS Comput Biol. 2021 Aug 17;17(8):e1009278. doi: 10.1371/journal.pcbi.1009278 (PMC8396727; doi:10.1371/journal.pcbi.1009278)
Supplement: Attachment — Submitted filename: CPA - comments 2.docx [file pcbi.1009278.s014.docx]

Reviewer #1: The authors have made an extensive revision to the original article(s). They have responded in a satisfactory way my questions and requests for clarification, as well to other reviewers’ comments. I have only a couple of comments that I hope can be incorporated in a final version of this article. These proposed changes do not require an additional revision.

1. The authors responded in a clear way potential use cases and applications of the database (Question 8), but I did not see this information incorporated in the final text. A little bit of this response is incorporated in Data availability but it is not complete. The authors should make sure to incorporate this information as it provides a guideline to general audiences about the importance of this work. A partial incorporation that helps retain style and continuity would be ok with me.

Thanks a lot for your suggestion. We have now included the applications of the CPAfold database in the results section.

2. Make sure the correct symbol is used for the biological hydrophobicity scale, at the moment the symbol appears as a broken symbol in the PDF.

Thanks for pointing out this error. We have now incorporated the symbol of the biological hydrophobicity (ΔG) scale in Figure 9.

Reviewer #2: All my comments and suggestions were addressed what is much appreciated. In my opinion, the merged manuscript reads very well (both the results and methods sections). I have just a few minor suggestions:

Thanks

Pg. 4. HHpred is not a tool for homology modeling but for template identification.

Thanks for the comment. We have added the homology modelling step that was performed with Modeller.

Pg. 4. “Most importantly, we identify a novel fold, named the reentrant-helix-reentrant fold, present in three families and describe their evolutionary history.” – I suggest adding a sentence giving a glimpse on what is new/interesting/unusual in the new fold (lack of symmetry?).

Thanks for the suggestion we have added the following line:

“The novel fold is characterized by the fusion of two reentrant helices in a single region wherein the homologous resolved structures of the reentrant helices are separated in two symmetric domains. “

Figure 3b. Node labels are too crowded and thus hard to read. Perhaps the authors could label nodes with numbers and provide a legend?

We have changed the dimension of the labels and fixed the overlap between the labels.

Pg. 8. “The network in Figure 5 clearly shows the evolutionary relationship” – Figure 3, I guess? Please check the figures numbering.

It has now been corrected.

Pg. 11, point 4. Font type/size seems to be different

It has now been corrected.

Pg. 12. “can evolve independently,, shuffle” – two commas?

It has now been corrected.

Final thought: most of the presented evolutionary scenarios involve an intermediate state corresponding to a non-functional protein (lack of the active site, broken symmetry, etc.). I am wondering about the mechanism that enabled their “resurrection”. Paralogs with a similar function? Formation of inter-family hybrids?

This is an interesting problem, where we can only speculate. Certainly, paralogs are likely to be necessary to maintain some functions. However, it is neither necessary to assume that the intermediates are non-functional. It is possible that the “new reentrant” region in PSE might exist as a minor state even in a full-length ancestral protein. We added a comment about this.
